# Effectiveness of Voice Therapy in Telepractice with Patients with Hyperfunctional Voice Disorder: A Pilot Study

**DOI:** 10.3390/jcm13175320

**Published:** 2024-09-08

**Authors:** Nayeon Choi, Suna Park, Gil Joon Lee

**Affiliations:** 1 Department of Otorhinolaryngology-Head and Neck Surgery, Samsung Medical Center, Sungkyunkwan University School of Medicine, Seoul 06351, Republic of Korea; chlskduschoi@naver.com; 2 Department of Otorhinolaryngology-Head and Neck Surgery, Kyungpook National University Chilgok Hospital, Kyungpook National University School of Medicine, Daegu 41944, Republic of Korea; nayeon.choi@samsung.com

**Keywords:** telepractice, digital treatment, voice disorder, voice therapy

## Abstract

**Background**: The need for telepractice and digital treatment has increased due to issues this revision.regarding medical access and the COVID-19 pandemic. However, in many countries, telepractice is rarely performed. The purpose of this pilot study was to describe the detailed process of telepractice in patients with hyperfunctional voice disorder and investigate its effects. **Methods**: The three subjects who were enrolled in this pilot study had hyperfunctional voice disorders. The evaluation was performed face to face. Auditory perceptual evaluation, acoustic evaluation, aerodynamic evaluation, patient self-evaluation, and interviews were conducted. Treatment was delivered by telepractice using a smartphone application. **Results**: In quantitative analysis of auditory perceptual evaluation, acoustic evaluation, aerodynamic evaluation, and patient self-evaluation, all subjects showed improved voice after treatment. In-depth analysis of telepractice was performed through the interview. **Conclusions**: Telepractice was effective in patients with voice disorders, and the patients were satisfied with this approach. In addition to this pilot study, further large-scale studies are required, but telemedicine may improve treatment outcomes and patient satisfaction in cases where medical access is limited or during outbreaks of respiratory infections like COVID-19.

## 1. Introduction

Voice disorder refers to an abnormality in vocal sound caused by a problem in respiration, phonation, or resonance, which are the steps involved in producing vocal sounds. This may cause psychological withdrawal and limitations in physical, emotional, and social activities of individuals, ultimately leading to a decline in quality of life [1]. Because voice is a part of expressing oneself and an important factor in social activities, there is an increasing demand for voice therapy for vocal improvement.

The main types of voice disorders that require voice therapy are functional and organic voice disorders, which are known as hyperfunctional voice disorder, caused by vocal nodules, vocal polyps, and muscle tension dysphonia [2]. Most patients diagnosed with hyperfunctional voice disorder, including teachers, salespersons, counselors, singers, and announcers, usually have an occupational voice disorder. While there is no dispute about the fact that voice therapy for such patients can be effective for vocal improvement and symptom recovery [3,4,5], many patients do not benefit from its therapeutic effects due to early discontinuation of treatment [3]. Therefore, regular treatment is needed to verify the effects of voice therapy, but there are limitations due to time and space constraints.

Some countries, apart from Korea, have already implemented telepractice services that can increase access to treatment, while also enabling efficient time management and resolving distance constraints. Such services have provided opportunities to patients who have recognized the need for treatment but have had difficulties attending a medical institution due to various circumstances. Telepractice has been steadily growing since the early 2000s [6], and as of the end of 2019, the need for telepractice has increased worldwide due to the COVID-19 pandemic.

The spread of COVID-19 involves airborne droplets that are released when an infected person coughs, sneezes, or talks, and they enter the respiratory system of another person, or infection occurs when a person touches an object that an infected person has touched [7]. Speech therapy, which requires the support of visual, auditory, and tactile cues, poses inevitable risks of infection transmission. Moreover, evaluation and treatment of patients with voice disorder presents a high risk of exposure to the virus due to the high likelihood of droplet discharge [8]. Accordingly, speech-language pathologists (SLPs) coming into face-to-face contact with patients were advised to keep at distance of at least 1.8 m from the patient or use plexiglass as a barrier and keep interactions with patients to 10 min or less; SLPs were also advised to conduct telepractice sessions [8]. In addition, voice therapy guidelines in the context of the COVID-19 pandemic recommended that each clinician must attempt to mitigate the risk of infection and achieve the best therapeutic results, considering the patient’s particular reality [8,9]. Furthermore, COVID-19 also influenced voice rehabilitation after head and neck cancer surgery, which could be solved using telepractice [9].

Several studies have investigated the effects of voice therapy using telepractice. Voice therapy has traditionally targeted young patients and adults who are competent in handling devices in the early years, although the target population has since expanded to include children and elderly persons [10,11]. One study reported that when patients with muscle tension dysphonia were divided into face-to-face and non-face-to-face treatment groups, both groups showed improvement in voice quality and quality of life [12]. Another study compared face-to-face and non-face-to-face treatment in patients with head and neck cancer who underwent voice therapy and swallowing therapy and reported that treatment was more effective and patient satisfaction was greater in the non-face-to-face group [13]. Therefore, the effectiveness of voice therapy using telepractice has been sufficiently validated, and while telepractice was just one of the options for the delivery of speech therapy before the COVID-19 pandemic, it has now become a key method for providing services that patients need [8].

However, voice therapy using telepractice has not been generally accepted in all countries. For example, Western European countries, such as Italy and France, recognize the need for telepractice, but have not been able to expand such services for a number of reasons [14]. Firstly, most hospitals are not equipped with basic equipment, such as cameras or software, needed for telepractice. Secondly, SLPs are not fully confident about telepractice. Thirdly, elderly or low-income individuals may not be able to purchase the electronic devices required for telepractice. Lastly, social medical systems and private insurance companies do not recognize telepractice as a procedure covered by insurance.

Korea also faces similar challenges. Despite the increased need for non-face-to-face treatment throughout society, many issues need to be considered and resolved before voice therapy using telepractice becomes universally accepted. However, the potential risk of exposure to infection is a major reason for implementing telepractice. Furthermore, telepractice enables the provision of appropriate services to patients who do not have access to required services due to time and distance constraints.

Accordingly, the present study aimed to conduct an in-depth analysis of voice therapy using telepractice by examining the therapeutic effects of such therapy on patients with hyperfunctional voice disorder and through self-reporting in an interview format by participants undergoing therapy. The main purpose of our study was to evaluate the feasibility of telepractice as a treatment for voice disorders. In addition, the study also aimed to assist many SLPs in their efforts to implement voice therapy using telepractice.

## 2. Materials and Methods

### 2.1. Participants

This pilot study included three patients diagnosed with hyperfunctional voice disorder by an otolaryngologist based on laryngoscopic findings among patients who visited the department of otolaryngology at a tertiary hospital. Because this pilot study had a small number of subjects, we performed quantitative comparisons of voice parameters before and after voice therapies.

Inclusion criteria were as follows: (1) patients with hyperfunctional voice disorder among patients with voice problems due to vocal fold lesions that were identified by laryngoscopy; (2) patients with no vision or hearing problems; (3) patients who have no difficulty using a mobile phone or computer; (4) patients with no previous history of voice therapy; and (5) patients who had not been treated with medication for voice problems. Patient information is presented in Table 1.

### 2.2. Procedures

The present study was conducted between October 2020 and July 2021, and the treatment period varied for each participant (Participant A: October 2020 to July 2021; B: April to June 2021; and C: May to July 2021). Vocal evaluations were performed at the hospital, and telepractice was implemented within 1 week of pre-treatment evaluation. The participants took part in the therapy, consisting of one 30-min session per week, at home using the TalkyTalky application. The number of therapy sessions was 6, 9, and 9 for Participant A, B, and C, respectively. The post-treatment evaluation was performed 1 week after the completion of therapy.

The therapist delivered the instructions using an LG GRAM notebook (15ZD980-GX50K) (LG Electronics, Seoul, Republic of Korea) and a USB microphone (CM-700USB). (LG Electronics, Seoul, Republic of Korea). 

Participants A and B used a notebook, while Participant C used a smartphone.

### 2.3. Evaluation Tools

#### 2.3.1. Auditory-Perceptual Evaluation

The GRBAS scale was used for auditory–perceptual evaluation of the participant’s voice. A grade 1 speech therapist with 10 years of experience performed the evaluation by listening to vowel sounds (ah, ee, and oo) and the first two sentences in a paragraph about “taking a walk”.

#### 2.3.2. Acoustic Evaluation

A multi-dimensional voice program (MDVP) and voice range profile (VRP) from the Computerized Speech Lab(Lincoln Park, NJ, USA) (CSL; Model 4500B, Kay Pentax) were used to evaluate the pitch, loudness, and quality of the participant’s voice. The MDVP was used to measure the fundamental frequency (F0), jitter, shimmer, and noise-to-harmonic ratio (NHR). With respect to the testing method, the microphone was placed 10 cm away from the mouth of the participant and fixed at 90°. The participant made the “ah” vowel sound three times, for least 5 s each, and the 1.5 s that best reflected the participant’s voice was selected and used in the analysis. A VRP was used to identify the vocal range. With respect to the testing method, the participant was instructed to make the “ah” vowel sound from the lowest to the highest note and from the highest to the lowest note. 

#### 2.3.3. Aerodynamic Evaluation

A Phonatory Aerodynamic System (PAS; Model 6600, Kay Pentax) (Lincoln Park, NJ, USA)was used to evaluate the respiration and phonation ability of the participants, measuring the maximum phonatory time (MPT), the mean airflow ratio (MAFR), and the subglottal pressure (Psub). For the measurement of the MPT and the MAFR, a rubber mask was placed tightly against the mouth and nose, after which the participant inhaled as much air as possible and made the “ah” vowel sound in a comfortable pitch and loudness. This process was repeated three times, and the longest measured result was used. For the measurement of the Psub, a rubber mask with a silicon tube attached was placed tightly against the mouth and nose, after which, the silicon tube was placed on top of the tongue. The participant made a “pa, pa, pa, pa, pa” sound three times. The first and last sounds were excluded, and the uttered sounds in the middle were used.

#### 2.3.4. Patient Self-Evaluation

The voice handicap index (VHI) was used for the self-evaluation of the participants own voices. The VHI consists of functional, physical, and emotional domains, with 10 items per domain for a total of 30 items. Each item was evaluated on a 5-point scale (0–4 points), with lower scores indicating higher satisfaction.

#### 2.3.5. Qualitative Evaluation

To supplement insufficiencies in quantitative evaluation, an interview-based qualitative evaluation was performed, which consisted of items for identifying satisfaction in voice therapy using telepractice, the advantages and disadvantages of voice therapy using telepractice, areas of improvement for voice therapy using telepractice, the intent to participate in future voice therapy using telepractice, and the preference between voice therapy using telepractice and face-to-face voice therapy.

### 2.4. Voice Therapy Program

The program consisted of holistic voice therapy technique with the goal of producing normal voice, focusing on the coordination between respiration, phonation, and resonance and voice facilitating techniques for correcting faulty vocal habits. Although the treatment goals were the same, the effects, adherence, and preference varied between the participants. Accordingly, different treatment contents were applied to different participants. The contents included vocal hygiene, laryngeal massage, abdominal breathing, yawn-sigh, the semi-occluded vocal tract exercise (SOVTE), and the vocal function exercise (VFE). Table 2 shows the contents applied and the homework assigned to each participant.

### 2.5. Telepractice Application

The present study used the TalkyTalky application to conduct the voice therapy using telepractice. The TalkyTalky application has a program Zoom installed in it to enable real-time telepractice. Moreover, when the therapist assigned treatment-related homework during each session, the participant submitted the tasks performed in audio or video format, and the therapist provided feedback [15].

### 2.6. Data Processing

Multi-dimensional vocal evaluations were performed to investigate the effects of voice therapy using telepractice, while qualitative evaluation was also performed to enhance the understanding of quantitative evaluations. Quantitative evaluations were performed before and after treatment, while the qualitative evaluation was performed upon completion of voice therapy. For the interview-based qualitative evaluation, a camcorder was used to record the utterances of Participants B and C, while a Galaxy 10 model smartphone was used to record the telephone interview with Participant A. The average length of the interview was 16.6 min (14 min 20 s–19 min 24 s), while the analysis of the interview was an average of six pages, referencing the method from a previous study [16].

## 3. Results

Participant A, B, and C received 6, 9, and 9 sessions of voice therapy, respectively, and each session took 30 min. All participants performed the tasks presented by the SLPs, and the SLPs gave feedback before the next treatment session when the task was uploaded as a video.

### 3.1. Auditory–Perceptual Evaluation

After the delivery of voice therapy using telepractice, all participants showed a decrease in GRBAS scores compared to pre-treatment, with improvement in hoarseness, roughness, and breathiness. The specific scores for each participant are presented in Table 3.

### 3.2. Acoustic Evaluation

After the delivery of voice therapy using telepractice, all participants showed higher F0 than at pre-treatment, and such increase was particularly prominent in Participant C. All participants also showed decreases in the MDVP parameter values (jitter, shimmer, and NHR), compared to pre-treatment. The VRP had expanded, and the specific results for each participant are presented in Table 4.

### 3.3. Aerodynamic Evaluation

After voice therapy using telepractice, all participants showed an increase in the MPT compared to pre-treatment. Participant A showed insufficient MAFR before treatment, although the MAFR was within the normal range after treatment. Participant B showed high Psub before treatment, although the Psub was within the normal range after treatment. The specific scores for each participant are listed in Table 5.

### 3.4. Patient Self-Evaluation

After the delivery of voice therapy using telepractice, all participants showed lower scores in the functional, physical, and emotional domains. The specific scores for each participant are listed in Table 6.

### 3.5. Qualitative Evaluation

#### 3.5.1. Patient Satisfaction with Voice Therapy Using Telepractice

Before commencing therapy, all participants had doubts about whether voice therapy using telepractice would be as effective as face-to-face therapy. Upon completion of the therapy, all participants were satisfied with voice therapy using telepractice.

Participant A

“I don’t have much spare time to go to receive treatment. Nonetheless, I wanted the treatment because of very bad discomfort in my neck. Telepractice … I thought it may be just a waste of time, but I was wrong. It was so convenient for me since I can receive treatment with just a computer or mobile phone. I can try the things I learned during the treatment at home, and the therapist gave me feedback, which ultimately was very helpful to me”.

Participant B

“I gained confidence from receiving the voice therapy. I started working again starting from three weeks ago. My voice still becomes hoarse if I talk too much at once or am near an air conditioner for a long time. But, by learning the vocal hygiene method that the therapist taught me and trying each one of the therapy methods, I feel like it’s getting better. It was so new. It was fascinating to do something with a computer or mobile phone, like my kid. Although it started because of COVID-19, I think it would be good to see it become more universal”.

Participant C

“I think it’s perfect for this social distancing era. Certainly, I should go to a hospital for in-person treatment, but I felt it was burdensome due to the distance. Actually, I wondered whether this would be effective before I started the therapy, but it has been very helpful since I can concentrate better from receiving the treatment at the most comfortable place”.

#### 3.5.2. Advantages and Disadvantages of Voice Therapy Using Telepractice

The advantages mentioned included efficient use of time and treatment taking place at a desired location. The disadvantages varied depending on the situation faced by each participant.

Participant A

“The best part was being able to receive the voice therapy in my spare time while working. I had the feeling of being managed by uploading what I did at home by voice or video recording. However, that part was somewhat annoying too. Also, I could not hear the therapist’s voice sometimes”.

Participant B

“Personally, I used to get ready early since the hospital is far from my house, but it was good to have time to spare. I was reluctant to go to the hospital because of COVID-19, but it was great that I can receive treatment without any sense of anxiety. However, it was too bad that how the treatment was being carried out could not be observed in detail, which the treatment with the therapist took place by video. I tried abdominal breathing for the first time. I did it as instructed by the therapist, but I’m not confident about whether I did it properly”.

Participant C

“The biggest advantage was efficiency with regard to time and space. Visiting a hospital to receive treatment was, in itself, a time burden, and travel was inconvenient, but not having such inconvenience and being able to receive treatment in space that I feel most comfortable in allowed me to concentrate better. However, the sound was not relayed sometimes due to the limitations in the audio function”.

#### 3.5.3. Areas of Improvement for Voice Therapy Using Telepractice

Technical issue in delivering voice therapy using telepractice was the common problem cited by the participants.

Participant A

“Even if I was just a little late to the treatment time set by the therapist, I had difficulty logging on. In such cases, the therapist helped me by sending a text or KakaoTalk message to restart the session. However, that took some time and I couldn’t do it in one attempt, which I thought was inconvenient”.

Participant B

“There were times when the screen froze, or the sound cut off during the session. That part should be upgraded”.

Participant C

“There was no problem with interaction, even though it was telepractice. However, when making sustained vowel sounds or vocalizing using a straw, sound came out well at first but faded after a while. I think the audio function should be improved, and I hope future sessions could be carried out in high definition”.

#### 3.5.4. Intent to Participate in Future Voice Therapy Using Telepractice

All three participants indicated that they would participate in future therapy sessions. Participant responses reflected the main aim in telepractice of avoiding potential infection due to COVID-19 and achieving efficient time management.

Participant A

“Of course, I would participate. I believe telepractice is the right thing to do in the current situation. During treatment, splashing of saliva is unavoidable, but by doing it at home, you can receive treatment comfortably without such worry. I believe telepractice is a perfect method of providing service in the current era”.

Participant B

“I was uncomfortable about going to the hospital, but it’s convenient that I don’t have to worry about that. I will definitely participate in telepractice if given another opportunity in the future”.

Participant C

“If given an opportunity, I do want to participate. I am much more comfortable talking than before the treatment. Even if I wanted to receive treatment, I often could not because it was too far, and (I) didn’t have the time, but if the treatment is in telepractice format, I can participate without any burden”.

#### 3.5.5. Telepractice vs. Face-to-Face Treatment

All participants preferred telepractice over face-to-face treatment. Because of the COVID-19 pandemic period, face-to-face medical consultations and social activities were restricted. Although they did not have experience of face-to-face treatment, they reported a satisfactory therapeutic effect from telepractice, and telepractice offered other benefits. Therefore, in their view, face-to-face treatment was not absolutely necessary.

Participant A

“I will choose telepractice. I have not received face-to-face treatment, but if I am satisfied this much with telepractice, then I don’t really need to have face-to-face treatment. Because of COVID-19, I’ve been avoiding contact with other people, so I am reluctant to take part in face-to-face treatment”.

Participant B

“Telepractice is better. Face-to-face treatment might be good too, but it is difficult to go to the hospital every time. Also, there are many other patients at the hospital, so there may be some unknown risks, so I think telepractice is more suitable for me”.

Participant C

“I think most would prefer telepractice. I also feel that way. I understood everything the therapist taught me, and it was easy to follow, even though the treatment was by telepractice. Although there were system errors, such as with the audio, it wasn’t enough to interfere with the treatment. More than that, being able to receive treatment at home and having time to spare were the biggest benefits for me”.

## 4. Discussion and Conclusions

In the present study, we conducted voice therapy using telepractice on patients with hyperfunctional voice disorder and identified vocal improvement based on multi-dimensional vocal evaluations. In addition, interview-based qualitative analysis was performed for the in-depth analysis of voice therapy using telepractice. The findings of the study were as follows.

### 4.1. Identification of Vocal Improvement through Vocal Evaluations

According to the GRBAS scale, which was used for auditory–perceptual evaluation, the results showed decreases in values for G, R, and B parameters. According to most previous studies, voice therapy using telepractice showed improvement based on auditory–perceptual evaluation [3,10,11], which is consistent with the findings of the present study. Therefore, voice therapy using telepractice showed a positive effect based on auditory–perceptual evaluation.

In the acoustic evaluation, the results showed an increase in F0 and decreases in jitter, shimmer, and NHR. Before treatment, the F0 of Participants A and C was lower than that of typical adult females. However, after treatment, the F0 increased to indicate vocal improvement. It is suspected that the F0 increased as a result of a reduction in vocal cord weight due to the reduction or removal of lesions through treatment. Moreover, decreases in jitter, shimmer, and NHR also indicated improvement in voice quality. Such findings were consistent with those of a previous study reporting a significant decrease in jitter and shimmer in patients with vocal polyps after applying multi-voice therapy techniques [4]. Although the treatment was not performed face to face like the previous study, the present study reconfirmed the effectiveness of voice therapy using telepractice by demonstrating the same improvement in voice quality. Additionally, VRP results showed expanded pitch range, which was also reported in a study in which patients with hyperfunctional voice disorder underwent a vocal aerobic treatment program [17]. In the present study, the pitch range expanded, particularly at high frequencies, which may be explained by two reasons. Firstly, treatment that induced relaxed vocalization improved vocal function to a normal level, and as a result, the F0 increased, while the vocalizable high-frequency range also expanded. Secondly, among the therapeutic techniques applied to the participants, VFE focused on gliding. Accordingly, because stretching training was carried out while controlling all laryngeal muscles and improving flexibility, such training was able to induce high-pitched sound to be produced naturally.

In the aerodynamic evaluation, the MPT increased, while the MAFR and the Psub appeared within the normal range after treatment. This can be viewed as improvement from enhanced respiration after voice therapy using telepractice. A case study on application of voice therapy using telepractice on children with voice disorder caused by septic pharyngitis and flu reported that the MPT increased from 6.7 to 15 s after 12 sessions of voice therapy [10]. Resonant voice therapy, VFE, and SOVTE were applied, and after treatment, the MPT increased, while hoarseness and strained voice were relieved. As in the previous study, the holistic voice therapy used in the present study focused on coordination between respiration, phonation, and resonance to promote proper vocalization. Consequently, increase in the MPT and improvement in voice quality were achieved.

Meanwhile, Participants B and C showed MAFR within the normal range regardless of treatment. However, Participant A showed an MAFR of 30 mL/s before the treatment, which increased to 80 mL/s after treatment, which was within the normal range. This result could be interpreted as the treatment promoting vocalization with the goal of relaxation being able to bring positive change to vocal function by relieving tension. Moreover, Participant C showed a decrease in the Psub from 13.98 cmH2O before treatment to 8.11 cmH2O after treatment, which was within the normal range. This result can also be interpreted as treatment with the goal of relaxation being able to improve the usual habit of uttering words forcefully and with tension.

In the patient self-evaluation using the VHI, participants showed lower scores in functional, physical, and emotional domains. It is suspected that participants felt that their voice had improved after voice therapy using telepractice. Previous studies have also reported that patients felt that the severity of their vocal problems were reduced after voice therapy using telepractice [3,17,18]. In particular, Participants A and B showed decreases of at least 22 points in their scores for the physical domain, and as a result, they showed a much greater margin of decrease than Participant C, showing a difference of at least 47 points in their overall scores. The severity of voice disorder perceived by each person is different. Participants A and B, who were occupational voice users, responded more sensitively to vocal changes than Participant C, who was a non-occupational voice user. Both Participants A and B perceived their vocal problems to be more serious than Participant C before treatment, and thus, they accepted the vocal changes after therapy using telepractice to be more significant. In other words, such findings could be understood as occupational voice users showing more positive acceptance of the effects of voice therapy using telepractice.

### 4.2. Interview-Based Qualitative Analysis

Firstly, the participants in the voice therapy using telepractice had doubts about the effectiveness of telepractice before starting treatment. However, they expressed satisfaction upon completion of treatment. The common factor that was satisfactory for all participants was not having to get ready for treatment since patients did not need to allocate separate time for voice therapy, and there were no space constraints, which means that accessibility to treatment was increased. In Korea, telepractice was initially adopted to eliminate the risk of COVID-19 infection, although once such practice was implemented, time and cost savings and greater access to treatment were viewed positively. From the patient’s perspective, satisfaction levels were high since vocal improvement could be achieved without the effort required to take part in face-to-face treatment. It is important to emphasize the elimination of potential viral infection and increased treatment access to promote the implementation of telepractice.

Secondly, all participants reported efficient time management as an advantage of voice therapy using telepractice, and additionally, Participant B indicated a positive evaluation of the reduced risk of COVID-19 spread. Participant C indicated that it was easier to concentrate since treatment was carried out in a comfortable place, consistent with reports in a study on voice therapy using telepractice for children [10]. During voice therapy for children, the guardian must watch from behind or observe from a distance, but with voice therapy using telepractice, the child can participate in the treatment together with his or her guardian at home. This can naturally build rapport between the therapist and the child and his or her guardian, while the child can comfortably take part in the treatment. Moreover, because treatment takes place at home, the treatment contents may be accepted more readily, and the transition to daily life may be easier.

However, technical problems were mentioned as a disadvantage of voice therapy using telepractice. The participants reported the audio and video not being in sync, or the audio cutting off or fading at times, which are the same problems encountered in other countries where telepractice is more prevalent [3]. At times, the participants had to reconnect after losing their internet connection during treatment, or sessions had to be cancelled due to difficulties with reconnection. It was suggested that an appropriate level of technical expertise is required to address such issues and that guidelines for telepractice need to be established. To resolve such technical difficulties, SLPs must coordinate with experts, and the participation of academic societies or associations for technical research and development should also be considered.

Another disadvantage mentioned was that there were parts that were difficult to understand within the treatment contents. Voice therapy using telepractice involves the exchange of information through the screen of the device used by each person. Generally, only auditory and visual cues are provided, but there are times when additional senses are required. Participant B expressed difficulty with sustained vocalization through abdominal breathing. Although the therapist explained how much the stomach should be expanded when inhaling and what physical changes are required when exhaling, it was not easy to perform the task. If the task was performed after personally feeling and comparing the physical changes in the other person, the treatment content may have been easier to understand. During telepractice, the therapist must provide accurate and detailed information to the patient, while the patients must try their best to accept such information. In addition, it is believed that the further development of treatment content and other related contents could be helpful in learning and generalizing the treatment content.

Thirdly, the areas for improvement of voice therapy using telepractice shared the same context as the technical limitations mentioned as the disadvantages of voice therapy using telepractice. In addition to technology development, institutional measures regarding telepractice must be established at the national level to promote the expansion of telepractice and change the perception of patients and therapists who use telepractice. Such actions could lead to universal acceptance of telepractice, which could naturally expedite the development of related technologies.

Fourthly, all participants indicated that they intend to participate in future telepractice, if given the opportunity. They mentioned prevention of concerns for potential COVID-19 infection and efficient time management. When vocal evaluations were classified according to the risk of exposure to COVID-19, self-evaluation by the patient or therapy using telepractice were viewed as low risk, while EGG or acoustic evaluation while wearing a mask was viewed as moderate risk, and aerodynamic evaluation was viewed as high risk [8]. It was reported that vocal evaluation must take place in the face-to-face setting since such evaluation is difficult to perform by telepractice, but voice therapy should be performed by telepractice to avoid the risk of infection. Moreover, because face-to-face treatment involves both the therapist and patient wearing a mask, there are limitations in relaying accurate information due to distorted pronunciation, while certain exercises, such as SOVTE, may be difficult to perform accurately [14]. Accordingly, voice therapy using telepractice was emphasized as a method that can overcome such issues. As described above, voice therapy using telepractice represents the present-day situation and it could be considered a method of providing beneficial service during potential future epidemics.

Fifthly, the participants preferred voice therapy using telepractice over face-to-face voice therapy. If the study had used an alternating treatment design to compare voice therapy using telepractice and face-to-face voice therapy, more reliable results may have been obtained. However, the participants were skeptical about the effectiveness of voice therapy using telepractice before their participation but were satisfied upon completion. From this, it can be inferred that patients prefer voice therapy using telepractice over face-to-face voice therapy. The participants were much more satisfied than they had anticipated since they noticed vocal improvement during voice therapy using telepractice and were able to comfortably participate in the treatment at the location of their choice while also being able to use their time efficiently. In particular, Participant B mentioned that she would like to see voice therapy using telepractice become more popular, and thus, it would be valid to view telepractice not just as an alternative to face-to-face treatment but as an equivalent method of service delivery.

Based on the findings described above, the significance of the present study can be found in the fact that it was the first study to identify vocal improvement through voice therapy conducted by non-face-to-face videoconferencing by providing only auditory and visual cues, when the discipline of speech therapy requires various cues, including auditory, visual, and tactile cues. Certainly, there are other studies in Korea that have reported on vocal improvement through voice therapy using telepractice [17,18]. However, the previous studies reported vocal improvement after applying both telepractice and face-to-face methods. Therefore, it is difficult to accept their findings that improvement was solely the effect of voice therapy using telepractice. However, all treatment sessions in the present study were conducted by telepractice and confirmed the usefulness of voice therapy using telepractice through vocal improvement, which can be viewed as important research findings. Moreover, by conducting an interview-based qualitative analysis with the participants, in-depth examination was also possible. However, it is difficult to generalize the findings due to the small sample size. Moreover, if the effectiveness of telepractice was demonstrated through a comparative study with a control group, the study could have been more meaningful.

Furthermore, the study selected patients with hyperfunctional voice disorder who have vocal fold lesions. Accordingly, the size of the lesion, the symmetry during vocal fold adduction/abduction, and the vocal fold vibration pattern of each participant could have been checked, but such factors were not analyzed after the treatment. Presenting the results from the analysis of such factors together could have enhanced the understanding of the effects of voice therapy using telepractice.

It would be useful to conduct future case studies on the speech therapists who delivered telepractice in the present study. Considering that other studies have reported that therapists with more experience tend to be more conservative toward telepractice [19] and that improvement in awareness among therapists is needed for the expansion of telepractice [14], a more specific approach is possible to determine which parts need improvement and upgrading for the expansion of telepractice.

In this post-COVID-19 era, people are using telepractice for some sense of comfort from the threat of infection. It is hoped that the findings of the present study will contribute to changing the perception about telepractice and provide an opportunity for telepractice to be incorporated into our lives so that therapy using telepractice does not remain a second-best choice but is regarded as a service that both SLPs and patients request as their first choice. As our research is a pilot study, it has limitations in drawing definitive conclusions. Therefore, validation through studies with a larger sample size and more rigorous statistical analysis is necessary.

## Figures and Tables

**Table 1:** Clinical characteristics of enrolled patients.

	Sex	Age	Occupation	Diagnosis	Duration of Symptoms
A	Female	28	Kindergarten teacher	Polyp	3 months
B	Female	51	Salesperson	Nodule	9 months
C	Female	35	Housewife	Nodule	1 month

**Table 2:** Treatment contents and homework for hyperfunctional voice disorder.

	Session	Contents	Homework
A	1	Vocal hygiene	Remembering vocal hygiene
		Laryngeal massage	5 times a day
	2~3	Yawn-sigh	5 times a day
	4~6	VFE	5 times a day
B	1	Vocal hygiene	Remembering vocal hygiene
		Laryngeal massage	5 times a day
	2~3	Abdominal breathing	10 times a day
	4~6	SOVTE	3 times a day for 5 min
	7~9	SOVTE	2 times a day for 5 min
		+VFE	3 times a day for 5 min
C	1	Vocal hygiene	Remembering vocal hygiene
		Laryngeal massage	5 times a day
	2	Abdominal breathing	3 times a day
	4~7	SOVTE	2 times a day
		+Yawn-sigh	3 times a day
	8~9	VFE	3 times a day

Abbreviation: SOVTE—semi-occluded vocal tract exercise; VFE—vocal function exercise.

**Table 3:** GRBAS before and after treatment.

		A	B	C
G	pre	1	2	1
	post	0	1	0
R	pre	1	1	1
	post	0	0	0
B	pre	1	1	1
	post	0	0	0
A	pre	0	0	0
	post	0	0	0
S	pre	0	0	0
	post	0	0	0

**Table 4:** MDVP and VRP before and after treatment.

		A	B	C
F0 (Hz)	pre	191.762	219.525	188.890
	post	215.292	222.980	230.344
Jitter (%)	pre	1.088	1.290	1.782
	post	0.326	0.630	0.264
Shimmer (%)	pre	3.505	4.390	3.185
	post	2.001	2.122	1.176
NHR	pre	0.138	0.198	0.152
	post	0.087	0.094	0.092
Voice Range Profile (HZ)	pre	138.59~349.23	155.56~440.00	143.85~341.85
	post	116.54~698.46	174.61~569.99	159.54~554.04

**Table 5:** PAS before and after treatment.

		A	B	C
MPT (s)	pre	10.13	7.50	14.81
	post	13.70	12.06	15.65
MAFR (mL/s)	pre	30	110	100
	post	80	80	120
Subglottal pressure (cmH_2_O)	pre	9.84	13.98	6.81
	post	7.66	8.11	7.99

**Table 6:** VHI before and after treatment.

		A	B	C
VHI-F	pre	22	29	18
	post	9	3	4
VHI-P	pre	26	28	14
	post	4	8	11
VHI-E	pre	18	22	10
	post	7	1	0
VHI-Total	pre	66	79	42
	post	20	12	15

## Data Availability

Data regarding this study could be provided by the corresponding author if there is a reasonable request.
